# Is the NH_4_^+^-induced growth inhibition caused by the NH_4_^+^ form of the nitrogen source or by soil acidification?

**DOI:** 10.3389/fpls.2022.968707

**Published:** 2022-09-09

**Authors:** Feng Wang, Qiang Wang, Qiaogang Yu, Jing Ye, Jingwen Gao, Haitian Liu, Jean W. H. Yong, Yijun Yu, Xiaoxia Liu, Haimin Kong, Xinhua He, Junwei Ma

**Affiliations:** ^1^Institute of Environmental Resources and Soil Fertilizers, Zhejiang Academy of Agricultural Sciences, Hangzhou, China; ^2^Department of Biosystems and Technology, Swedish University of Agricultural Sciences, Alnarp, Sweden; ^3^School of Biological Sciences, The University of Western Australia, Perth, WA, Australia; ^4^Arable Soil Quality and Fertilizer Administration Station of Zhejiang Province, Hangzhou, China; ^5^Centre of Excellence for Soil Biology, College of Resources and Environment, Southwest University, Chongqing, China

**Keywords:** soil acidification, ammonium stress, nitrogen assimilation, wheat growth, natural variation

## Abstract

Soil acidification often occurs when the concentration of ammonium (NH_4_^+^) in soil rises, such as that observed in farmland. Both soil acidification and excess NH_4_^+^ have serious adverse effects on crop growth and food production. However, we still do not know which of these two inhibitors has a greater impact on the growth of crops, and the degree of their inhibitory effect on crop growth have not been accurately evaluated. 31 wheat cultivars originating in various areas of China were planted under 5 mM sole NH_4_^+^ (ammonium nitrogen, AN) or nitrate nitrogen in combined with two pH levels resembling acidified conditions (5.0 and 6.5). The results showed that the shoots and roots biomass were severely reduced by AN in both and these reduction effects were strengthened by a low medium pH. The concentration of free NH_4_^+^ and amino acids, the glutamine synthetase activity were significantly higher, but the total soluble sugar content was reduced under NH_4_^+^ conditions, and the glutamine synthetase activity was reduced by a low medium pH. Cultivar variance was responsible for the largest proportion of the total variance in plant dry weight, leaf area, nodal root number, total root length and root volume; the nitrogen (N) form explains most of the variation in N and C metabolism; the effects of pH were the greatest for plant height and root average diameter. So, soil acidification and excess NH_4_^+^ would cause different degrees of inhibition effects on different plant tissues. The findings are expected to be useful for applying effective strategies for reducing NH_4_^+^ stress in the field.

## Introduction

The industrial nitrogen (N) fertilizers application has contributed tremendously to the increase in global food production (Gong et al., 2011; Zhang et al., 2013). However, less than half of the applied N (30–40%) is utilized by crops in China to date (Ding et al., 2020). The N lost included run-off, volatilization, leaching, and denitrification, which together lead to a range of major ecological challenges and environmental problems (Chen et al., 2014). Unlike nitrate nitrogen (NN), ammonium (NH_4_^+^) nitrogen (AN) can be assimilated into amino acid production by plants without further chemical reduction (Coskun et al., 2017). The NH_4_^+^-based stabilized fertilizers application was considered to be a promising future strategy for improving agronomic N use efficiency (Ren et al., 2021). Unfortunately, most dryland crops, including wheat, can be markedly inhibited when the concentration of ammonium in soil is too high, whereas the comparable inhibitory effect does not occur for NN fertilization (Miller and Cramer, 2005; Coskun et al., 2017). In southern China, especially in red-yellow soil areas, approach 20 mM NH_4_^+^ concentration in soil was recorded (Xu et al., 2012), much higher than the 0.1–0.5 mM optimal NH_4_^+^ concentration for upland crops (Liu et al., 2013).

The membrane potential would be depolarized during the NH_4_^+^ uptake and assimilation process, resulting in an increase in the net proton release, as well as external soil acidification (Zhu et al., 2012). Over the last few decades, the Ca^2+^, Mg^2+^, and K^+^ depletion and sulfur (S) and N atmospheric deposition have promoted soil acidification and reduced the soil’s buffering capacity, particularly in the red-yellow soil of southern China, which is located in tropical/subtropical regions; in this region, an average soil pH of 4.5–5.5 has been recorded (Guo et al., 2010). Soil acidification also produces adverse effects on cell metabolism (Van Den Berg et al., 2005). NH_4_^+^ was assimilated into organic N compounds in cells primarily through the GS/GOGAT pathway (glutamine synthetase, GS, EC 6.3.1.2; glutamate synthase, GOGAT, EC 1.4.1.14) (El Omari et al., 2010; Xu et al., 2012). Plant species maintaining a low intracellular with higher GS activity are thought to be more tolerant to NH_4_^+^ toxicity (Cruz et al., 2006). In addition, the increased carbon consumption caused by increased NH_4_^+^ assimilation in roots also partially explain NH_4_^+^-induced growth inhibition (Britto and Kronzucker, 2002). However, few studies have examined the relationship between NH_4_^+^ stress and soil acidification or whether the NH_4_^+^-induced growth inhibition is caused by an NH_4_^+^ form source or soil acidification caused by NH_4_^+^ absorption.

The understanding of plant growth in response to NH_4_^+^ tolerance is mainly based on studies of mutants and transformants or some model plants such as rice, *Arabidopsis* et al. (Guo et al., 2007; Zhu et al., 2009; Li et al., 2010). Wheat is one of the most widely cultivated crops in the world, which is sensitive to NH_4_^+^ toxicity (Wang et al., 2019). In many cases, wheat have to face excess NH_4_^+^ in China, which is often the case in the field with the use of slow-release fertilizers and nitrification inhibitors, especially during fertilization stag. It is a very important and necessary object to study genetic variability of wheat adaptation to NH_4_^+^ toxicity combined with soil acidification. Its ecotypes have been found show a wide range of habitats differing notably in soil richness. These natural variations indeed provide a framework to study the adaptation of different traits that define plant growth in relation to simultaneous genetic changes and environmental variations, while there is lack of studies on this topic In this study, we conducted hydroponic experiments using 31 wheat cultivars in media with different N forms (AN or NN) and pH values. The objectives of this study were to evaluate the inhibitory degree of NH_4_^+^ form source and soil acidification on crop growth. The results would provide useful insight into the selection and breeding of crop cultivars with greater potential to adapt to NH_4_^+^-form N source combined with soil acidification in the field.

## Materials and methods

### Plant materials and experimental design

Seeds of 31 wheat varieties of China selected from a collection that was shown to maximize the genetic diversity of a large set of genotypes of China (Supplementary Table S1) were used. 20% (v/v) H_2_O_2_ were used to surface-sterilized uniform seeds for 10 min, and then rinsed the seeds more than 5 times with distilled water. The seeds were germinated on filter paper covering with wet sterilized gauze, and then transplanted them to sterilized silica sand until the seedling bud length was about 1 cm. At two-leaf stage, uniform seedlings were transplanted into black plastic containers (length × width × height, 45 cm × 32 cm × 15 cm) in an artificial illumination incubator with a 16 h/8 h light (a 1,200 μmol m^−2^ s^−1^ photosynthetic photon flux density)/dark with temperatures of 18°C day/10°C night cycle. A modified Hoagland nutrient solution (Gao et al., 2018) was used with two N form sources: sole NO_3_^−^ source solution (NN) or a sole NH_4_^+^ source solution (AN). The sole NO_3_^−^ source solution composition was as follows: macronutrients (mmol L^−1^): 5.0 N as KNO_3_ and Ca(NO_3_)_2_ or (NH_4_)_2_SO_4_, 3.0 K as KH_2_PO_4_ and K_2_SO_4_ or KNO_3_, 1.5 Ca as CaSO_4_, or CaCl_2_ and Ca(NO_3_)_2_, 1.0 Mg as MgSO_4_, 1.0 P as KH_2_PO_4_, 0.5 Na as NaCl; and micronutrients (mmol L^−1^): 1.0 Fe as Fe-EDTA, 0.15 × 10^−3^ Zn as ZnSO_4_, 0.16 × 10^−3^ Cu as CuSO_4_, 9.10 × 10^−3^ Mn as MnSO_4_, 18.5 × 10^−3^ B as H_3_BO_3_, 0.52 × 10^−3^ Mo as MoO_3_. The sole NH_4_^+^ source solution composition was as follows, macronutrients (mmol L^−1^): 5.0 mM N as (NH_4_)_2_SO_4_, 3.0 mM K as K_2_SO_4_ and KH_2_PO_4_, 1.5 mM Ca as CaSO_4_ and CaCl_2_, 1.0 mM P as KH_2_PO_4_, 1.0 mM Mg as MgSO_4_, and 0.5 mM Na as NaCl. The micronutrients composition in the AN source solution were the same with those in the NN source solution.

The pH of the AN or NN source solution was adjusted to 5.0 (to imitate the acidified red-yellow soil region of southern China) or 6.5 (as the normal condition for wheat growth) by adding 0.1 mM H_2_SO_4_ or 0.1 mM NaOH to the solutions; thus, the solutions are denoted by the treatment solution-pH as NN-6.5, AN-6.5, NN-5.0, and AN-5.0. The pH of the Hoagland solution was adjusted daily to the pH 5.0 or 6.5 also using 0.1 mM NaOH or 0.1 mM H_2_SO_4_ during the whole experimental process. Dicyandiamide (A nitrification inhibitor, 1 mM) was added to prevent NH_4_^+^ microbial oxidation. The solutions were replenished every 3 days. All treatments had three replicates with a completely random design.

### Plant sampling and traits analysis

Shoots and roots were harvested 21 days after the start of the different pH values and N forms treatment, frozen in liquid nitrogen, the stored at −80°C for physiological analysis, and dried partially at 105°C for 20 min and then 75°C for 24 h to obtain a constant weight. Root morphology was estimated from images of plants taken by a root scan machine (Epson 1,680, Indonesia), with WinRhizo Pro Vision 5.0 analysis program (Canada). A Li-3000 area meter (Li-Cor Inc., Lincoln, NE, United States) was used to measure leaf area. Free NH_4_^+^ concentration in plant shoot and root was measured according to the method of Balkos et al. (2010). Soluble sugars concentration in tissues were measured from the hydroalcoholic extracts using a colorimetric assay with an anthrone reagent (Wang et al., 2019). Free amino acids concentration in shoot and root was determined using the method of Moore and Stein (1954). GS activity and nitrate reductase (NR) activity were determined, as described previously (Wang et al., 2016).

### Statistical analysis

The variance (ANOVA) analysis was carried out using the SPSS general linear model procedure (SPSS 19.0, United States). The different forms and pH levels of N nutrition and different varieties were considered the observed variation sources for the studied cultivars. The quantity of variation (%) for each factor was estimated using the total calculated sum of squares (Wang et al., 2016). The figures were created using SigmaPlot graphics software (ver. 14.0; Systat Inc., San Jose, CA, United States).

## Results

### Plant growth

**Figure 1:**
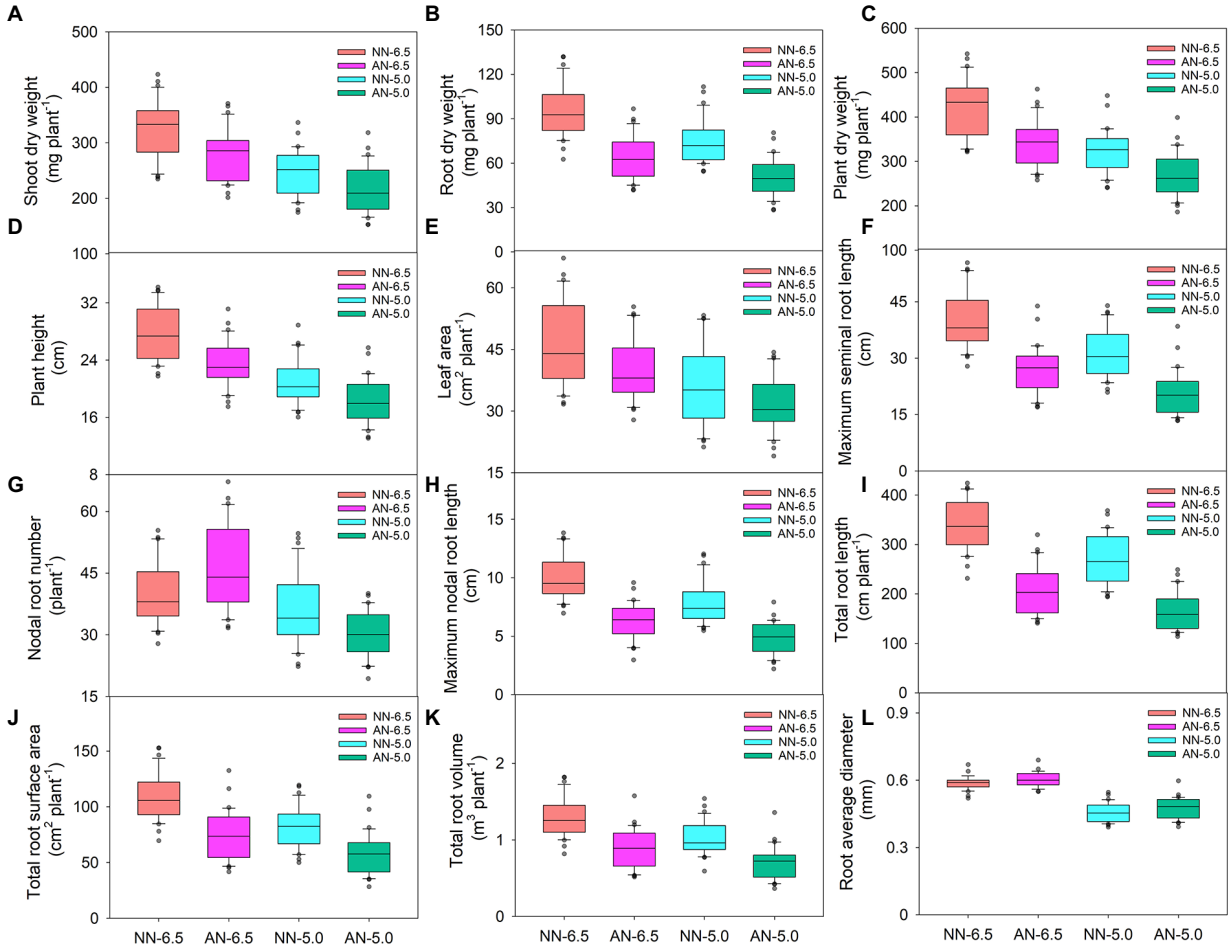
Biomass allocation, plant height, leaf area and root morphology of 31 wheat cultivars grown under different N-sources and pH levels. Data are means ± SE (n = 31). NN-6.5 refers to the nitrate nitrogen at a pH of 6.5; NN-4.5 refers to the nitrate nitrogen at a pH of 6.5; AN-6.5 refers to the ammonium nitrogen at a pH of 6.5; AN-5.0 refers to the ammonium nitrogen at a pH of 5.0.

Both the sole NH_4_^+^ source and rhizosphere acidification inhibited plant growth (Figure 1). Compared to normal growth conditions (NN-6.5, NN at a pH of 6.5), shoot, root, and plant dry weights, plant height; and leaf area were reduced by 14.7, 33.4, and 19.0%; 16.2%; and 13.0%, respectively, for AN-6.5; 23.7, 21.7, and 23.2%; 24.3%; and 23.3% for NN-5.0; and 33.3, 47.0, and 36.4%; 34.0%; and 31.7% for AN-5.0. All of these parameters significantly differed according to cultivar, N form, and pH level; also, there were N form × pH levels interaction effects (Table 1). In shoot dry weight, plant dry weight (41.4%), and leaf area (60.4%), cultivar variance explained the largest proportion of the total variance (48.1%). Differently, N forms and pH levels accounted for the largest proportion of the variance in root dry weight (40.3%) and plant height (38.2%), respectively.

**Table 1:** *F-*values in two-way ANOVA analysis of shoot dry weight, root dry weight, plant dry weight, plant height and leaf area in 31 wheat cultivars seedlings under different N-sources and pH levels.

Source	df	Shoot dry weight		Root dry weight		Plant dry weight		Plant height		Leaf area	
		Squares sum (×10^3^)	*F-*value	Squares sum (×10^3^)	*F-*value	Squares sum (×10^3^)	*F-*value	Squares sum (×10^2^)	*F-*value	Squares sum (×10^2^)	*F-*value
Cultivar	30	218 (48.1)	16^**^	22.9 (38.1)	17^**^	300 (41.4)	16^**^	9.5 (34.5)	8.6^**^	72.9 (60.4)	19.6^**^
pH	1	145 (32.1)	336^**^	8.6 (14.5)	202^**^	225 (31.1)	378^**^	10.6 (38.2)	286.5^**^	28.8 (23.9)	232.4^**^
N-forms	1	48 (10.7)	112^**^	24.2 (40.3)	565^**^	141 (19.5)	237^**^	3.9 (14.4)	108.1^**^	7.4 (6.2)	60.2^**^
pH × N-forms	1	2 (0.5)	4^*^	0.5 (0.8)	10^**^	4 (0.6)	7^**^	2.2 (0.9)	6.7^*^	0.35 (0.3)	2.8 ns
Error	90	39 (8.6)		3.8 (6.4)		53 (7.4)		3.3 (12.0)		12.4 (9.2)	

N-forms refers to the three nitrogen-forms effect; pH refers to the three pH levels effect; N-forms × pH refers to nitrogen-form × pH levels interaction effects. Values in brackets following the sum of squares for variables indicate the percentage (%) of given variation to total variation. ^*^ and ^**^ indicate significant at 0.05 and 0.01 level, respectively. df refers to the degree of freedom. ns means to no significant difference.

Root morphogenesis also depended on N form and rhizosphere acidification conditions (Figure 1). Compared with the NN-6.5 condition, maximum seminal and nodal root length, total root length, root surface area and root volume decreased by 33.4, 37.6, 38.3, 31.7 and 31.1% respectively, but nodal root number and root average diameter increased by 15.0 and 2.6%, respectively, at AN-6.5. Compared with NN-6.5, at NN-5.0, maximum seminal and nodal root length, nodal root number, total root length, root surface area, root volume, root average diameter decreased by 22.3, 21.7, 11.7, 20.8, 24.9, 20.3, 22.7% respectively, meanwhile, the decline was even greater at AN-5.0, which was decreased by 48.3, 37.6, 22.7, 51.2, 47.6, 45.5 and 19.7%, respectively. These traits also varied significantly according to cultivar, N form, pH level and N form × pH level interaction effects (Table 2). N forms accounted for the largest proportion of total variance in root length, and cultivar variance was the greatest for root number (59.4%), surface area (42.4%), and volume (47.0%). The average diameter of roots was significantly affected by pH level, but was not affected by the pH and N form interaction.

**Table 2:** *F-*values in two-way ANOVA analysis of root morphological traits in 31 wheat cultivar seedlings under different N-sources and pH levels.

Source	df	Maximum seminal root length		Nodal root number		Maximum nodal root length		Total root length		Total root surface area		Total root volume		Root average diameter	
		Squares sum (×10^2^)	*F-*value	Squares sum (×10^2^)	*F-*value	Squares sum	*F-*value	Squares sum (×10^4^)	*F-*value	Squares sum (×10^3^)	*F-*value	Squares sum	*F-*value	Squares sum (×10^3^)	*F-*value
Cultivar	30	42.2 (37.1)	12.8^**^	71.9 (59.4)	20.5^**^	258 (32.5)	10^**^	22.9 (28.8)	17.4^**^	38.6 (42.4)	12.4^**^	5.86 (47.0)	19.2^**^	0.113 (15.4)	4.9^**^
pH	1	17.1 (15.1)	156^**^	30.0 (24.9)	2574^**^	100 (12.6)	116^**^	10.2 (12.8)	233^**^	15.3 (16.8)	148^**^	1.55 (12.5)	153^**^	0.542 (74.0)	719^**^
N-forms	1	43.8 (38.5)	399^**^	0.18 (0.2)	1.5 ns	354 (44.6)	412^**^	42.2 (52.8)	963^**^	27.1 (29.7)	262^**^	4.10 (32.9)	403^**^	0.009 (1.2)	11.3^**^
pH × N-forms	1	0.67 (0.6)	6.1^*^	8.3 (6.9)	71.6^**^	4.3 (0.6)	5.1^*^	0.56 (0.7)	12.8^**^	0.73 (0.8)	7.1^**^	0.05 (0.4)	4.5*	0 (0)	0.05 ns
Error	90	9.8 (8.7)		10.5 (8.7)		77 (9.7)		3.9 (4.9)		9.3 (10.2)		0.91 (7.3)		0.68 (9.3)	

N-forms refers to the three nitrogen-forms effect; pH refers to the three pH levels effect; N-forms × pH refers to nitrogen-form × pH levels interaction effects. Values in brackets following the sum of squares for variables indicate the percentage (%) of given variation to total variation. ^*^ and ^**^ indicate significant at 0.05 and 0.01 level, respectively. df refers to the degree of freedom. ns means to no significant difference.

### Free NH_4_^+^ concentration

**Figure 2:**
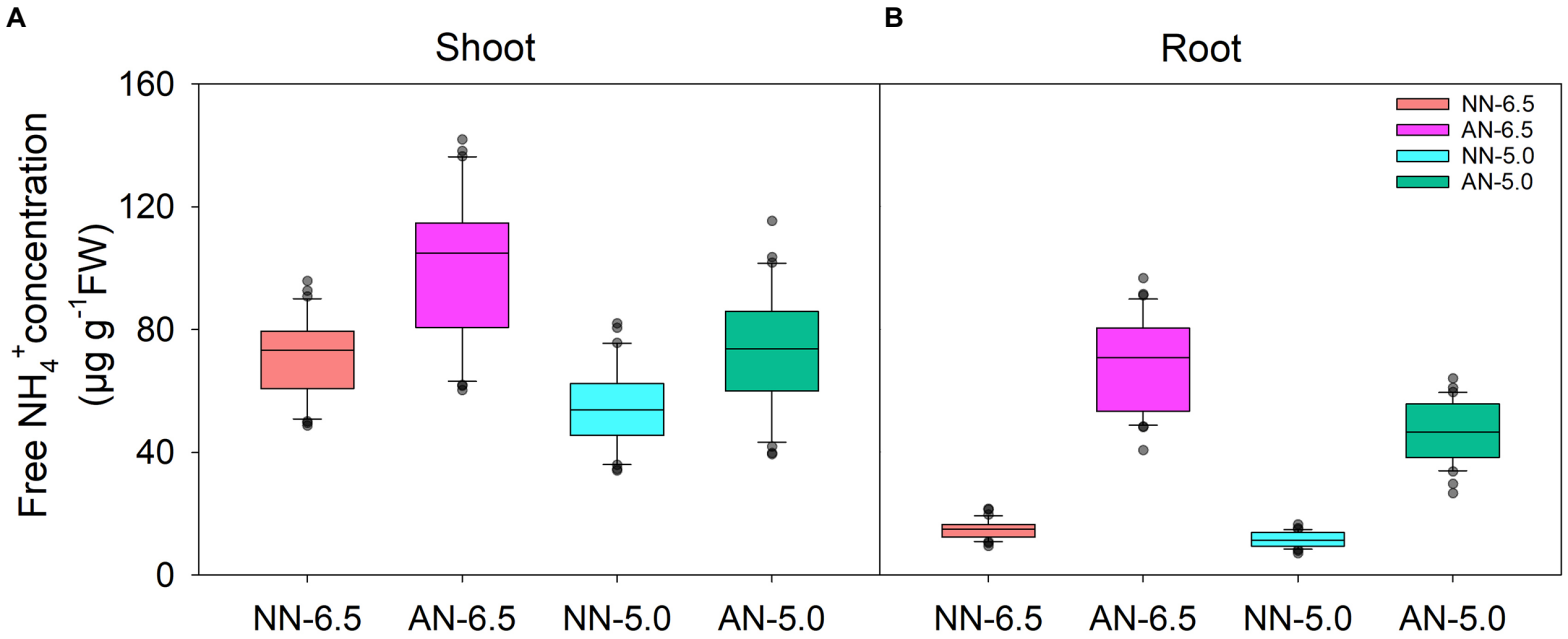
The levels of NH_4_^+^ in 31 wheat cultivars grown under different N-sources and pH levels. Data are means ± SE (n = 31). NN-6.5 refers to the nitrate nitrogen at a pH of 6.5; NN-4.5 refers to the nitrate nitrogen at a pH of 6.5; AN-6.5 refers to the ammonium nitrogen at a pH of 6.5; AN-5.0 refers to the ammonium nitrogen at a pH of 5.0.

The concentration of free NH_4_^+^ in wheat under AN was apparently higher than that under NN nutrition condition at a uniform pH (Figure 2). Compared to NN-6.5, the concentration in shoots was 42.2 and 2.1% higher under AN-6.5 and AN-5.0 but 22.5% lower under NN-5.0 treatments. It changed more in roots than in aerial parts. Compared to NN-6.5, the concentration in roots increased by 365.3 and 214.4% under AN-6.5 and AN-5.0 but decreased by 21.5% under NN-5.0 treatments. In shoot and root, the free NH_4_^+^ concentrations significantly varied by cultivar, N form, and pH level, but changes in cultivars caused the highest proportion of the variance (47.1%) in shoot; but N forms explained the largest proportion of total variance (77.0%) in root.

### Soluble sugar and free amino acid concentration

**Figure 3:**
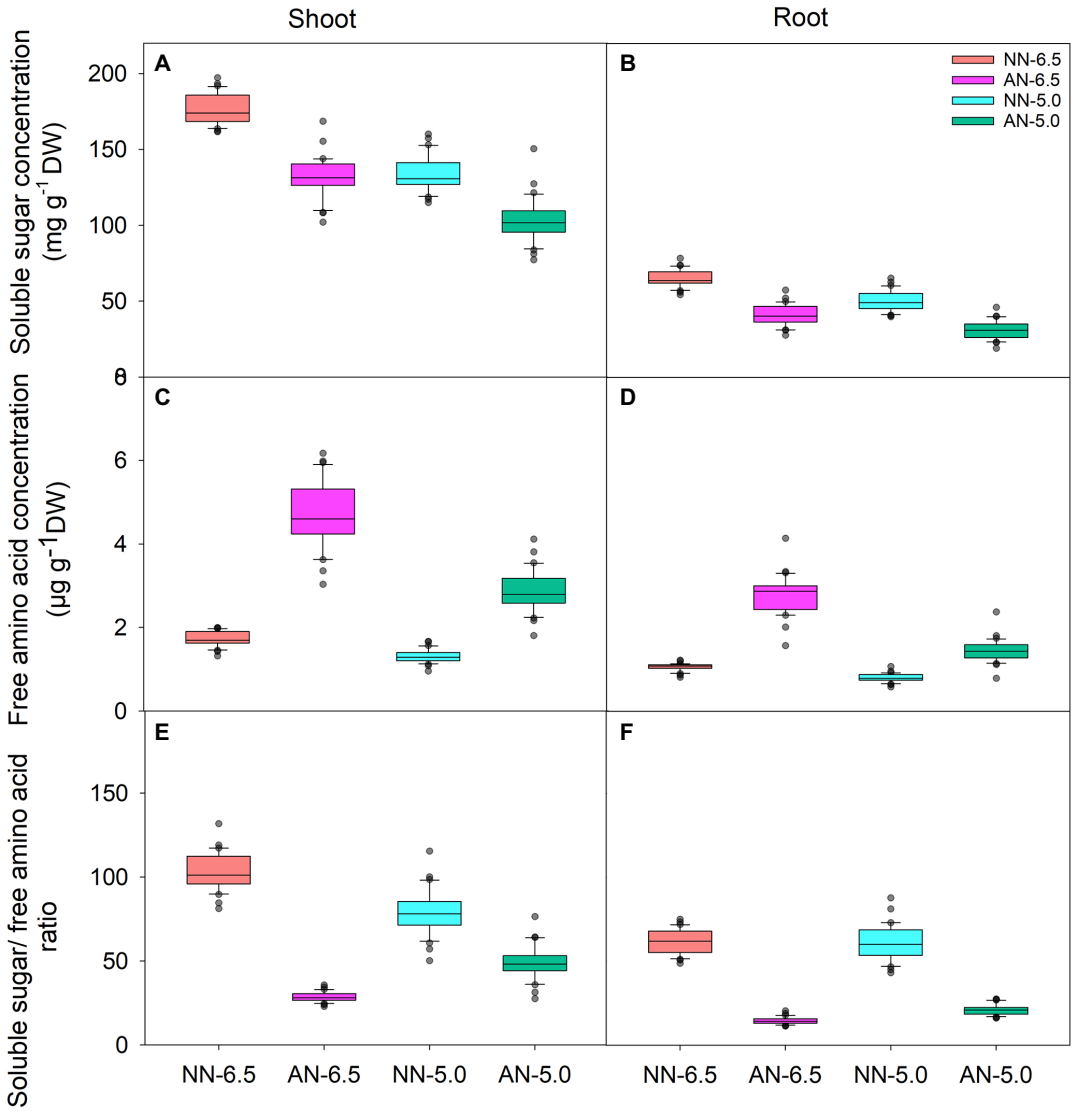
The levels of soluble sugar, free amino acids and soluble sugar/ free amino acid ratio in shoots and roots of 31 wheat cultivars grown under different N-sources and pH levels. Data are means ± SE (n = 31). NN-6.5 refers to the nitrate nitrogen at a pH of 6.5; NN-4.5 refers to the nitrate nitrogen at a pH of 6.5; AN-6.5 refers to the ammonium nitrogen at a pH of 6.5; AN-5.0 refers to the ammonium nitrogen at a pH of 5.0.

Soluble sugar, as the C skeleton for NH_4_^+^ assimilation, also varied with N form and rhizosphere acidification conditions (Figures 3A,B). Compared to NN-6.5, the soluble sugar concentrations in shoot and root were lower under AN-6.5, NN-5.0, and especially AN-5.0; also, the level of changed tended to be higher in roots than in shoots. In contrast, as the primary assimilation product of NH_4_^+^, free amino acid levels were significantly higher under AN than under NN nutrition. The concentration of free amino acids in shoots under AN-6.5 and AN-5.0 increased by 171.6 and 64.4%, respectively, compared to NN nutrition with pH 6.5, but decreased by 24.3% under NN-5.0 treatment, as shown in Figures 3C,D. Compared to NN-6.5, the concentration of free amino acids in roots under AN-6.5 and AN-5.0 increased by 161.9 and 38.0%, respectively, and was reduced by 22.8% in roots under NN-5.0. The ratio of soluble sugar to free amino acid reflects a balance status of C and N in the process of NH_4_^+^ assimilation. As shown in Figures 3E,F, compared to nitrate treatments, low pH and AN markedly reduced the ratio of soluble sugar of free amino acid in shoot and root. Compared to NN-6.5, NN-5.0, AN-6.5, and AN-5.0 showed reductions in the soluble sugar/free amino acid ratio of 72.3, 22.9, and 52.0% in shoots and by 76.1, 0.7, and 65.7% in roots, respectively. N form explained most of the variance in the soluble sugar and free amino acids concentrations in shoot and root (Table 3).

### GS and NR activities

**Figure 4:**
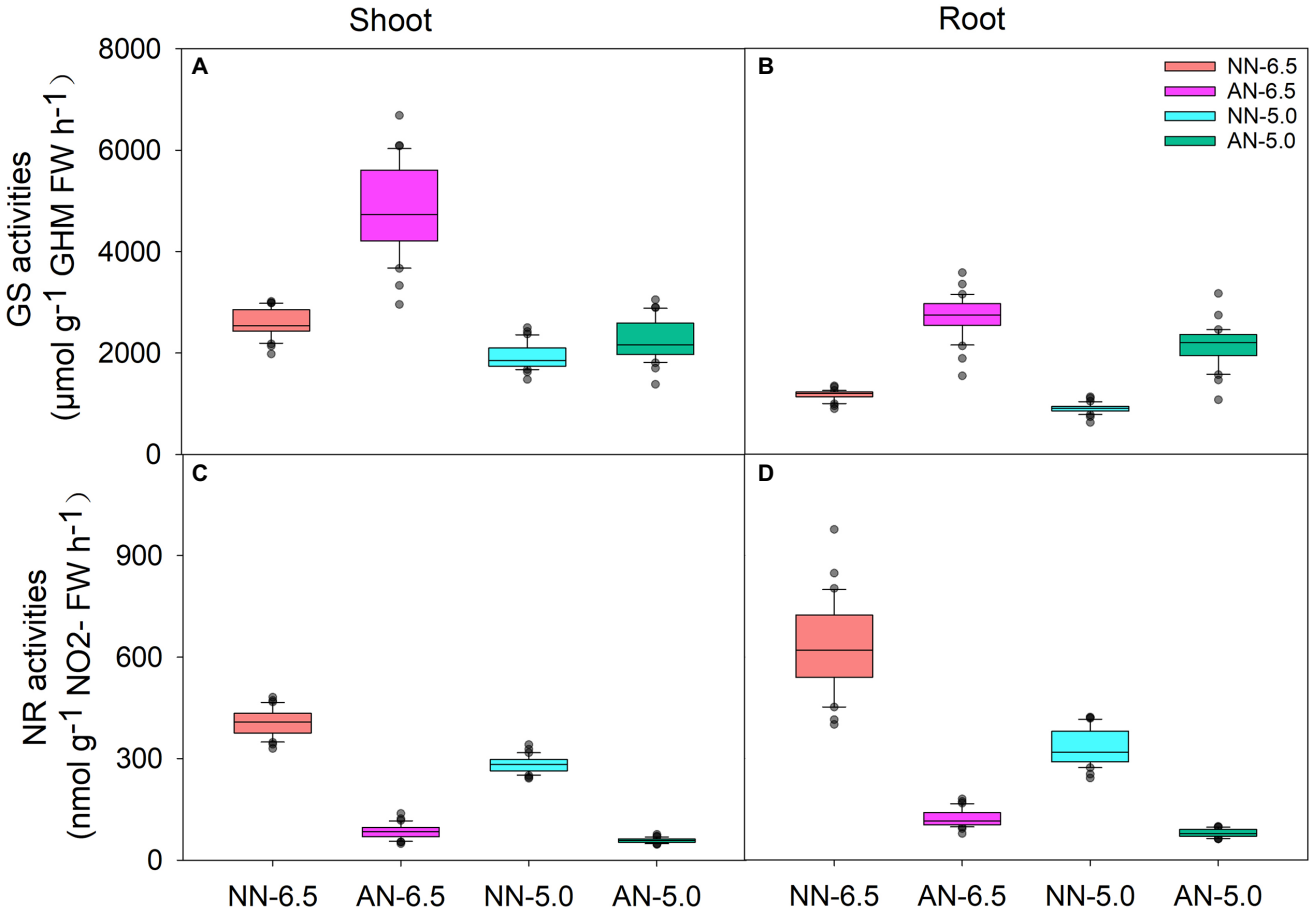
The enzymatic activities of GS and NR in shoots and roots of 31 wheat cultivars under different N-sources and pH levels. Data are means ± SE (n = 31). NN-6.5 refers to the nitrate nitrogen at a pH of 6.5; NN-4.5 refers to the nitrate nitrogen at a pH of 6.5; AN-6.5 refers to the ammonium nitrogen at a pH of 6.5; AN-5.0 refers to the ammonium nitrogen at a pH of 5.0.

GS and NR activities represent the N assimilation ability of wheat and significantly differed by cultivar, N form, and pH level (Figure 4). Compared to NN-6.5, the GS activity increased (86.8%) in shoots under AN-6.5 and decreased (25.2 and 12.8%, respectively) under NN-5.0 and AN-5.0; in roots, it increased (133.7 and 83.2%, respectively) under AN-6.5 and AN-5.0 but decreased (83.2%) under NN-5.0. Compared to NN treatments, pH and AN markedly reduced the NR activity in both shoots and roots. Their activities also varied apparently among all the cultivar, N forms, pH levels and the interaction effects of nitrogen-form × pH levels (Table 4). Variance in pH levels had the strongest effect on shoot GS activity (42.0%); N form had the greatest effect on the GS activity in root, and the NR activity in both shoot and root.

**Table 3:** *F-*values in two-way ANOVA analysis of GS and NR activities in shoot or root of 31 wheat cultivar seedlings under different N-sources and pH levels.

Source	df	GS activities in shoot		GS activities in root		NR activities in shoot		NR activities in root	
		Squares sum (×10^6^)	*F-*value	Squares sum (×10^6^)	*F-*value	Squares sum (×10^3^)	*F-*value	Squares sum (×10^6^)	*F-*value
Cultivar	30	21.7 (11.4)	6.7^**^	6.1 (8.0)	4.6^**^	31.9 (1.2)	1.9^*^	0.29 (4.6)	2.4^**^
pH	1	80.3 (42.0)	744^**^	5.6 (7.3)	126^**^	169 (6.5)	316^**^	0.87 (13.7)	221^**^
N-forms	1	50.7 (26.6)	470^**^	60.7 (78.5)	1361^**^	2,297 (87.9)	4297^**^	4.3 (68.7)	1108^**^
pH × N-forms	1	28.6 (15.0)	264^**^	0.8 (1.0)	18^**^	67.7 (2.6)	126^**^	0.47 (7.5)	120^**^
Error	90	9.7 (5.1)		4.0 (5.2)		48.1 (1.8)		0.35 (5.6)	

N-forms refers to the three nitrogen-forms effect; pH refers to the three pH levels effect; N-forms × pH refers to nitrogen-form × pH levels interaction effects. Values in brackets following the sum of squares for variables indicate the percentage (%) of given variation to total variation. ^*^ and ^**^ indicate significant at 0.05 and 0.01 level, respectively. df refers to the degree of freedom. ns means to no significant difference.

## Discussion

Several studies have provided a general description of plant responses to changes in the AN environment (Britto and Kronzucker, 2013; Wang et al., 2019); however, most of them have considered only a small number of genotypes, in which the effects of soil acidification caused by NH_4_^+^ absorption is neglected, thus only a partial view of the phenomena given. Since wheat ecotypes have been found in a wide range of habitats differing notably in NH_4_^+^ conditions in our previous studies (Wang et al., 2016, 2019, 2020), few studies have reported that using this natural variation among ecotypes as an alternative way to search genes related to NH_4_^+^ tolerance. In the present studies, the responses of wheat to different N forms and pH values was studied by measuring morphological traits and metabolic traits in 31 wheat genotypes. Result showed that cultivar variance explained the largest proportion of total variance in shoot dry weight, plant dry weight, leaf area, nodal root number, total root surface area, total root volume and the free NH_4_^+^ concentration in shoot when wheat seedlings were planted in the solution as NN-6.5, AN-6.5, NN-5.0 or AN-5.0. Previous studies found wheat was sensitive to increased NH_4_^+^ concentration in soil (Cao et al., 2009; Ariz et al., 2010; Zhu et al., 2012; Setien et al., 2013), furtherly, the present results clearly indicated that NH_4_^+^ nutrition and soil acidification significantly reduced biomass accumulation and leaf area in wheat plants. The dry weight of roots was considerably reduced by AN treatment, not only due to limitations in root emergence but also because of a reduction in root length (reduced by 22.3 and 21.7% in maximum seminal root length and nodal root length, respectively). This NH_4_^+^-induced inhibition is related to root growth and development also has been observed by some previous studies (Cramer and Lewis, 1993; Britto and Kronzucker, 2002; Zhang and Wang, 2012). Notably, in the present study, the decline was even greater at lower pH levels under AN conditions, but N form still accounted for the largest proportion of variance in root length (Table 2). Li et al. (2010, 2013) found that nearly 70% of the NH_4_^+^-induced root elongation inhibition was accounted by the cell expansion reduction, which root elongation and development, was mediated by reduced auxin by NH_4_^+^ condition (Li et al., 2011). In the present study, the nodal root number was maintained or increased under AN but decreased at lower pH. The nodal root number was more strongly affected by variation in pH than by variation in N form (Table 2), which might be explanted by a higher accumulation of cytokinin in NH_4_^+^-fed roots (Wang et al., 2019) in our previous study.

As a typical symptom of “ammoniacal syndrome,” free NH_4_^+^ concentration in plant cells was significantly higher under AN conditions than under NN. This may be associated with the ability of root system to absorb NH_4_^+^ (Zhu et al., 2009), the rate of NH_4_^+^ efflux from root inside (Li et al., 2011), and also NH_4_^+^ assimilation in root cell (Setien et al., 2013). The free NH_4_^+^ concentration increased under AN condition but decreased with lower pH in roots, but N forms was the crucial factor as Table 4 shown. The uptake of NH_4_^+^ depolarizes the plasma membrane, increasing the net proton (H^+^) release from the cell inside to rhizosphere soil (Zhu et al., 2009), furtherly leading to rhizosphere acidification. The H^+^-ATPase in plasma membrane made a decisive contribution to maintain the balance of pH inside and outside cells, which hydrolyzed ATP to pump H^+^ out; and an electrochemical gradient generated which could drive the free NH_4_^+^ ions active transport crossing the plasma membrane (Zhu et al., 2012). At a low medium root pH, the activity of H^+^-ATPase must be smartly enhanced to pump H^+^ outside to adapt to an increased electrochemical gradient, to ensure NH_4_^+^ uptake (Zhu et al., 2009). Therefore, the low medium pH may inhibit NH_4_^+^ absorption while high pH may facilitate NH_4_^+^ uptake (Brix et al., 2002). Our results also showed that, in wheat root, the N form was the main reason for the changes in the free NH_4_^+^ concentration which was consistent with the previous studies; however, interestingly, free NH_4_^+^ in wheat shoot varied most by cultivar, which had not been reported before.

**Table 4:** *F*-values in two-way ANOVA analysis of free NH_4_^+^ concentration, soluble sugar, free amino acid concentration and soluble sugar/ free amino acid ratio in shoot or root of 31 wheat cultivars seedlings under different N-sources and pH levels.

Source	df	Free NH_4_^+^ concentration in shoot		Free NH_4_^+^ concentrations in root		Soluble sugar concentration in shoot		Soluble sugar concentration in root		Free amino acid contents in shoot		Free amino acid contents in root		Soluble sugar/free amino acid ratio in shoot		Soluble sugar/free amino acid ratio in root	
		Squares sum (×10^3^)	*F-*value	Squares sum (×10^3^)	*F-*value	Squares sum (×10^3^)	*F-*value	Squares sum (×10^3^)	*F-*value	Squares sum	*F-*value	Squares sum	*F-*value	Squares sum (×10^3^)	*F-*value	Squares sum (×10^3^)	*F-*value
Cultivar	30	34.2 (47.1)	23^**^	6.5 (8.4)	5.3^**^	11.4 (11.0)	3.6^**^	3.2 (13.3)	6.2^**^	17.2 (7.2)	5.2^**^	5.6 (7.0)	4.6^**^	7.09 (6.3)	3.9^**^	3.1 (4.8)	4.1^**^
pH	1	15.3 (21.1)	318^**^	4.9 (6.3)	121^**^	37.9 (36.6)	366^**^	4.5 (18.6)	262^**^	38.2 (15.9)	346^**^	18.8 (23.4)	469^**^	0.05 (0)	0.9 ns	0.27 (0.4)	11.2^**^
N-forms	1	17.5 (24.1)	362^**^	60.4 (77.0)	1471^**^	43.4 (42.0)	420^**^	14.8 (60.9)	860^**^	160 (66.7)	1453^**^	43.3 (54.0)	1080^**^	84.3 (75.2)	1407^**^	58.6 (90.7)	2356^**^
pH × N-forms	1	1.2 (1.7)	24^**^	2.7 (3.6)	67^**^	1.4 (1.4)	13.8^**^	0.21 (0.9)	12.2^**^	14.7 (6.1)	134^**^	8.9 (11.1)	222^**^	15.3 (13.6)	255^**^	0.36 (0.6)	14.77^**^
Error	90	4.3 (6.0)		3.6 (4.7)		9.3 (9.0)		1.5 (6.4)		9.9 (4.1)		3.6 (4.5)		5.3 (4.8)		2.2 (3.5)	

N-forms refers to the three nitrogen-forms effect; pH refers to the three pH levels effect; N-forms × pH refers to nitrogen-form × pH levels interaction effects. Values in brackets following the sum of squares for variables indicate the percentage (%) of given variation to total variation. ^*^ and ^**^ indicate significant at 0.05 and 0.01 level, respectively. df refers to the degree of freedom. ns means to no significant difference.

Apart from the ability of plant root to absorb NH_4_^+^, the allocation of NH_4_^+^ also depends on the N metabolism process. NH_4_^+^ absorbed by roots is mostly assimilated into amino acids in root cells (Masclaux-Daubresse et al., 2010). In the present study, AN resulted in a remarkedly increase in the free amino acid concentration in shoots and roots, confirming a metabolic adaptation to AN condition, as previously discussed by Lothier et al. (2011). The enhanced N compound pool was probably due to both high NH_4_^+^ absorption and increased N assimilation to cope with having solely AN to maintain plant growth (Wang et al., 2020). The negative correlation between free amino acid concentration and soluble sugar concentration in roots and shoots was detected, and AN markedly reduced the soluble sugar/free amino acid ratio, indicating that more N-assimilation occurred in roots. Thus, a sufficient carbon skeleton supply for NH_4_^+^ assimilation is required under AN condition. A higher photosynthetic capacity increases plant NH_4_^+^ assimilation and tolerance, providing C skeletons to fuel the cell respiratory action and translocate more carbohydrates downward to maintain root growth (Ariz et al., 2011). In the present study, a distinct decrease in soluble sugar concentration accrued under AN condition (as the carbon skeleton for NH_4_^+^ assimilation) in 31 wheat cultivars, particularly in the roots (Figure 3); however, this decrease was lower at pH 6.5 than at pH 5.0, possibly due to the higher photosynthetic capacity at pH 6.5 (Ariz et al., 2011, 2013), and also the inhibited capacity of NH_4_^+^ assimilation. If the capacity of NH_4_^+^ assimilation is depressed by low pH, more NH_4_^+^ may accumulate in root and then be transported upward to the shoot, which is proven to be more sensitive to NH_4_^+^ stress (Britto and Kronzucker, 2002; Cruz et al., 2006); this leads to inhibition of photosynthesis and CO_2_ fixation (Guo et al., 2007), resulting in a further reduction in C assimilation.

Some studies have found that the relative assimilation enzyme activity in the GS/GOGAT cycle plays an important role for crops in adaptation to AN condition (El Omari et al., 2010). In the present study, GS activity was much increased, but NR activity was decreased by AN (Figure 4); pH level had the strongest effect on shoot GS activity and N form had the greatest effect on root GS activity. In different tissues of cells, GS exists as isoforms GS1 (found in the cell cytosol) and GS2 (in plastids) (Singh and Ghosh, 2013). GS1 is the predominant enzyme in non-photosynthetic tissues or root, but slightly presents in green tissues like young stem or leaf (Simon and Sengupta-Gopalan, 2010), which metabolic role includes assimilating NH_4_^+^ into amino acids (glutamine) for N transport and distribution (Bernard et al., 2008). AN treatment increased the GS activity, especially the root GS activity (the predominant isoform GS1). The increased capacity of NH_4_^+^assimilation by GS1 could prevent NH_4_^+^ accumulation in the roots and transport to the shoots (Setien et al., 2013), but this higher GS activity was not inhibited by the low medium pH significantly, indicating GS1 was insensitive to low pH. GS2 played important roles in NH_4_^+^ re-assimilation during photorespiration and the part of NH_4_^+^, which was derived from nitrate reduction (Singh and Ghosh, 2013), mostly located in the chloroplasts stroma, and its physiological importance has been demonstrated in transgenic plants overexpressing GS2 in leaves (Bernard et al., 2008). Increased GS2 activity had an enhanced capacity for photorespiration, also retained more than 90% of their photosystem II activity to fight against osmotic stress (Bernard and Habash, 2009). In the present study, a relative increase in shoot GS activity (GS2) may maintain photorespiratory capacity while improving the cultivar’s tolerance to NH_4_^+^ stress, but unlike GS1, its activity decreased sharply under low pH, and pH level had the strongest effect on it, indicating GS2 was more susceptible to soil acidification stress.

## Conclusion

**Figure 5:**
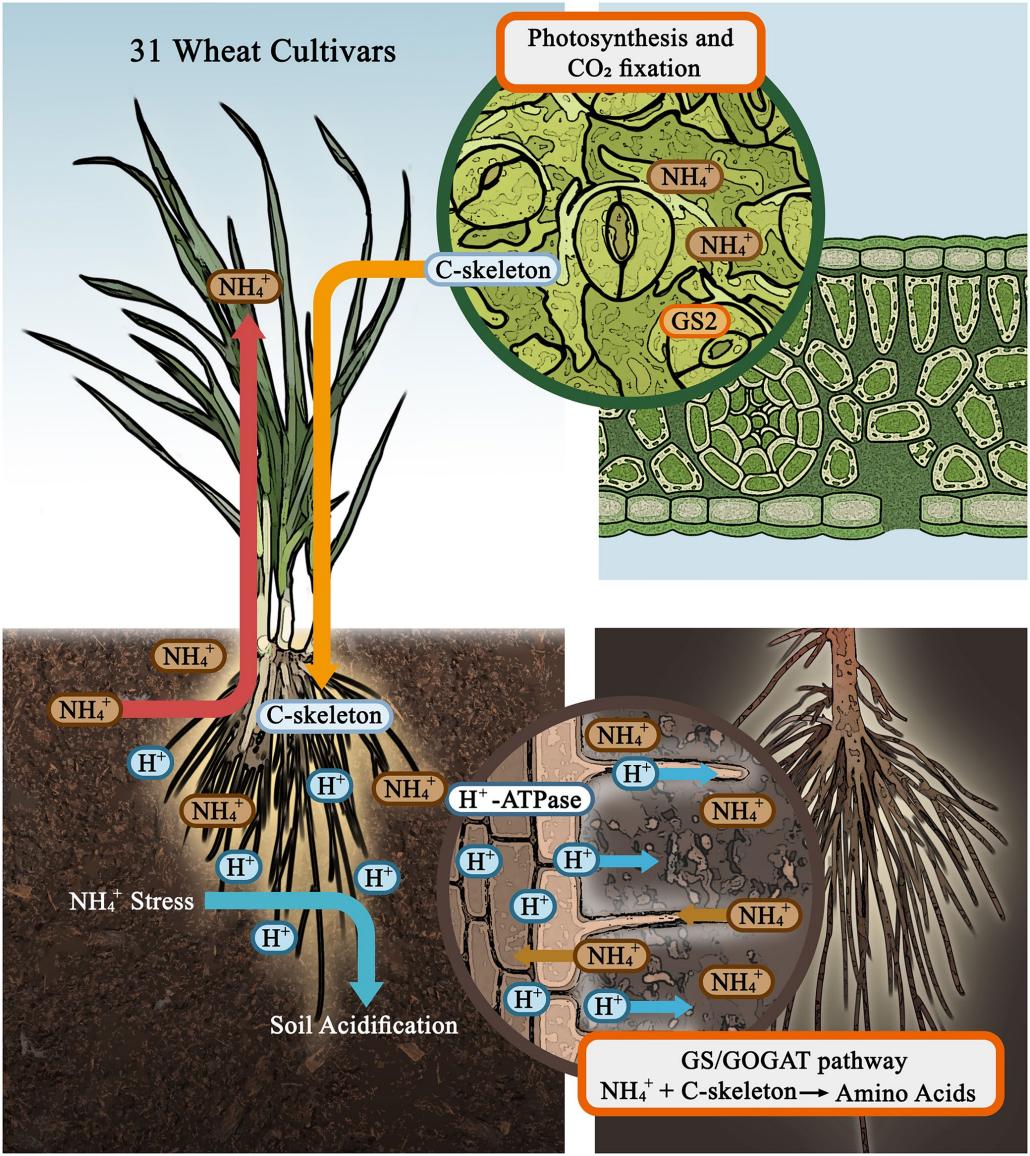
A proposed model of wheat physiological and biochemical responses to ammonium stress and soil acidification.

In conclusion, we studied the variation in N assimilation capacity of 31 wheat seedlings with respect to plant resistance to NH_4_^+^ stress and rhizosphere acidification (the model as shown in Figure 5). The growth of wheat seedling was seriously inhibited by AN, and this inhibitory effect was enhanced by soil acidification stress. Under AN, crops accumulated more NH_4_^+^ in tissues and more efficiently converted NH_4_^+^ into amino acids, and the N form accounted for the largest proportion of the variance in this process compared to the cultivar and pH level in root, but pH level had the strongest effect in shoot. To select and breed crop cultivars with a greater capacity to adapt to NH_4_^+^-N nutrition combined with soil acidification stress in the future, a higher capacity for N assimilation in both shoot and root and the ability to maintain high levels of C, must be a key focus.

## Data availability statement

The raw data supporting the conclusions of this article will be made available by the authors, without undue reservation.

## Author contributions

FW: experiments implementation, writing–original draft, resources, data curation, methodology, formal analysis, and visualization. JM: supervision and funding acquisition. QW, QY, JYe, JG, and HL: writing–original draft, data curation, methodology, formal analysis, and visualization. JYo and XH: conceptualization and writing–review and editing. YY, XL, and HK: investigation. All authors contributed to the article and approved the submitted version.

## Funding

We acknowledge generous financial support from the “Zhejiang Provincial Key Research and Development Program” (2020C02030 and 2021C02035); Project of leading talents of scientific and technological innovation in Zhejiang Province (2021R52045); Construction Project of Agricultural Green Development Pilot Support System of Huangyan District (HY202001); and Project of Unification of Water and Soil Science for One Healthy funded by the Zhejiang Academy of Agricultural Sciences.

## Conflict of interest

The authors declare that the research was conducted in the absence of any commercial or financial relationships that could be construed as a potential conflict of interest.

## Publisher’s note

All claims expressed in this article are solely those of the authors and do not necessarily represent those of their affiliated organizations, or those of the publisher, the editors and the reviewers. Any product that may be evaluated in this article, or claim that may be made by its manufacturer, is not guaranteed or endorsed by the publisher.
