# Supplementary material for: Is the NH4+-induced growth inhibition caused by the NH4+ form of the nitrogen source or by soil acidification?
Source: Front Plant Sci. 2022 Sep 9;13:968707. doi: 10.3389/fpls.2022.968707 (PMC9505920; doi:10.3389/fpls.2022.968707)
Supplement: Supplementary file 1 [file Table_1.DOCX]

Table S1. Wheat cultivars released at different areas tested in this experiment

| China Spring; Nanda2419; Huoshaotou; Bimamai; Xiaoyan4hao; You bao mai; Funo; Abbe; Jiang dong men; Fengchan3hao; Bodijiang; Yangmai1hao; Yumai13; Lumai 1hao; Yangmai 13; Yangmai 158; Ningmai 9hao; Zhengmai 9523; Zhengmai 9023; Yannong 19; Xuzhou25; Yangmai 11; Sumai 6hao; Lumai 15hao; Yumai 49; AK58; Huaimai 25; Ningmai 13; Zhenmai 5hao; Jinhe 9123; Yangmai 16 |
| --- |
